# Analysis of Carbon Metabolism Mechanisms and Reduction Strategies Toward Low-Carbon Steel Manufacturing

**DOI:** 10.3390/ma19132847

**Published:** 2026-07-03

**Authors:** Lei Zhang, Su Yan, Yuxing Yuan, Tao Du

**Affiliations:** Key Laboratory of Eco-Industry, Ministry of Ecology and Environment, Northeastern University, Shenyang 110819, China

**Keywords:** iron and steel manufacturing, carbon metabolic mechanism, carbon accounting, decarbonization strategy

## Abstract

**Highlights:**

- A mechanism-based carbon metabolism model integrating material and energy flows is developed.
- Carbon fixation, migration, and dissipation patterns of steel production are precisely quantified.
- Prioritized decarbonization strategies are matched to various processes through car-bon metabolic patterns.

**Abstract:**

Reducing emissions is increasingly critical for mitigating the environmental impact of the iron and steel industry. Achieving this transition requires an accurate evaluation of carbon emission intensity for steel production, which relies on an in-depth analysis of carbon metabolism mechanisms across the entire steel production chain. Existing approaches predominantly focus on carbon tracing within material flows, which cannot deeply integrate carbon migration pathways with energy flows and thus fail to reveal the actual sources and transmission mechanisms of carbon emissions. To address this gap, this study develops a carbon metabolism simulation model of the steel manufacturing process that considers the coupling of material production with the energy network. The differentiated carbon metabolism patterns are characterized in terms of carbon fixation, migration, and dissipation to support more accurate carbon emission accounting and enable the formulation of targeted decarbonization strategies. The results show that the coking process fixes 72.51% of its carbon input. The sintering and pelletizing process shows typical carbon dissipation characteristics, with nearly 100% of input carbon discharged. Carbon emissions from steelmaking and the rolling process are mainly induced by indirect energy consumption. The total carbon dissipation of integrated steel production system is 440.62 kg-C/t-CS, with the ironmaking process contributing the largest share of 33.92%.

## 1. Introduction

The iron and steel industry provides essential material sources for infrastructure, transportation, and manufacturing, but it is also the carbon-intensive industrial sectors, contributing 7% of global CO_2_ emissions annually [1,2]. The iron and steel sector will continue to face substantial carbon mitigation pressure because global steel demand is expected to remain high over the next 5–10 years. Global steel demand is projected to increase slightly by 0.3% to 1.724 billion tons in 2026 and then accelerate to 2.2% growth, reaching 1.76 billion tons in 2027 [3]. Crude steel (CS) capacity is expected to reach 1.97 billion tons in 2030 [4]. Although steel demand in some mature economies may stabilize or decline slightly due to slower construction activity, improved material efficiency, and recycling, demand in emerging economies is projected to experience 3–5% annual demand growth [5], driven by infrastructure construction, urbanization, and manufacturing expansion. With the accelerating climate crisis and the increasingly stringent mitigation targets established by international agreements such as the Paris Agreement, a rapid and fundamental shift toward low-carbon steel manufacturing has become unavoidable [6,7,8]. This profound transition to low-carbon production is not merely an environmental imperative but a strategic necessity for the industry’s long-term economic viability, competitiveness, and social license to operate under carbon constraint [9,10]. Iron and steel production combines diverse processes and energy conversion units, establishing strong couplings between heterogeneous material and energy flows, which affects the feasibility and effectiveness of reduction strategies. The development and implementation of effective decarbonization strategies require a deep, mechanistic understanding of carbon flows and transformations within the complex steel production processes [11,12].

The complexity of carbon metabolism in iron and steel manufacturing is profoundly influenced by the dominant production routes [13]. In integrated iron and steel plants, the traditional blast furnace-basic oxygen furnace (BF-BOF) route heavily relies on coke. Carbon serves important roles as a reducing agent for iron ore and as the primary thermal energy resources through combustion [1,14]. The complex reactions within the BF, involving the reduction of iron oxides by CO derived from solid fuels, inevitably lead to substantial CO_2_ emissions [15,16]. Carbon from coke is converted through coupled gasification, reduction, and oxidation processes. Adjustments in temperature, oxygen enrichment, and other process parameters can shift the partitioning of CO and CO_2_ [17], which further regulates the location and mechanism of carbon oxidation. Moreover, carbon can be partitioned into metal and slag through dissolution and reactions with slag components. Electric arc furnace (EAF) steelmaking primarily utilizes scrap steel and generates a comparatively lower carbon footprint attributable to electrodes, foaming agents [18], and supplementary heating sources. In hydrogen-based metallurgy [19], the carbon metabolic pathway is fundamentally altered because hydrogen serves as the primary reductant for iron oxides, thereby replacing coke as the dominant reduction agent [20,21]. It implies that the direct reduction product is predominantly water rather than CO or CO_2_ under sufficiently effective control of side reactions. A comprehensive understanding of the carbon transformation pathway is necessary. While numerous studies have quantified macroscopic carbon flows and overall emissions [22], a detailed mechanistic analysis explicitly linking specific operational parameters to the precise distribution and speciation of carbon transformation and its subsequent emission intensities remains lacking.

The existing carbon accounting approaches commonly applied to iron and steel production, including the IPCC tiered methods, life-cycle assessment, and input–output analysis, are principally designed to quantify total emission intensities. Zhang et al. [11] developed an input–output process simulation model and analyzed six iron and steel production routes combining conventional BF-BOF, EAF-based, and DRI-based configurations. A comparative analysis was performed on 22 hypothetical scenarios with different combinations of routes and hydrogen production measures. Wu et al. [23] provided a systematic review of carbon footprint calculation methods for the iron and steel industry based on LCA principles and demonstrated their application at the source, process, and end-use stages, as well as in cleaner production, through specific case studies. Zhang et al. [24] analyzed global iron and steel industry emissions over a 60-year historical period and forecast emissions up to 2050, evaluating CO_2_ alongside conventional and unconventional air pollutants. Wu et al. [25] presented an integrated emission inventory encompassing air pollutants and CO_2_ emissions from 811 iron and steel enterprises and five key manufacturing processes in 2020. While the existing tools are important for tracking emission levels, they are insufficient for addressing key internal process-level questions, including the extent to which carbon is incorporated into intermediate products, the pathways through which it migrates across processes, and the stages at which it is ultimately released as CO_2_. Moreover, these methods cannot reveal how such carbon partitioning responds dynamically to changes in material, energy inputs, and operational parameters. In particular, existing studies predominantly focus on carbon tracing within material flows. These approaches cannot integrate carbon migration pathways with the multi-energy network that inherently couple material production and energy conversion. Consequently, the sources and transmission mechanisms of carbon emissions remain obscured, making it more difficult to identify the most effective measures for deep decarbonization.

To address the formidable environmental challenges, multi-dimensional carbon reduction strategies are developed, which can be broadly categorized into process optimization [26,27], material and energy substitution [28,29], and the integration of breakthrough technologies [30,31,32,33]. Process optimization focuses on enhancing energy efficiency through waste heat recovery [34,35], process intensification, intelligent control [36], and improved resource utilization. Increased scrap steel utilization in BOF and EAF significantly reduces reliance on primary production, though limited by scrap availability and processing requirements. Carbon capture and utilization technologies [37] represent a critical pathway for mitigating emissions from existing, hard-to-abate processes by capturing CO_2_ before entering the atmosphere. However, CCU faces significant challenges related to capture efficiency, energy consumption, high capital and operational costs, and the development of robust transport, long-term storage, and more efficient utilization. A more transformative approach lies in breakthrough technologies to fundamentally alter the ironmaking paradigm [38]. Hydrogen-based metallurgy, in which green hydrogen replaces carbon as the primary reductant, holds considerable promise for achieving near-zero direct process emissions. However, the widespread adoption of hydrogen metallurgy necessitates massive investments in renewable energy infrastructure, water electrolysis for green hydrogen production, and plant construction, posing considerable economic challenges [39,40]. In addition, Trinca et al. [41] presents a techno-economic assessment of an innovative industrial-scale process for producing green steel from low-grade ores with net-negative CO_2_ emissions. This process includes a hydrometallurgical stage, where iron oxides are selectively dissolved by oxalic acid into ferric salts. Other revolutionary technologies encompass iron ore electrolysis and the employment of biomass and waste plastics as alternative reducing agents. Although a wide range of decarbonization technologies is available, effective emission reduction depends on matching technologies precisely to specific application scenarios. This requires a comprehensive understanding of carbon metabolism within the steel manufacturing process and its systemic impacts. Though extensive research has been conducted on macroscopic carbon flows, a critical knowledge gap remains: mechanistic analyses that quantitatively link specific processes and their material and energy inputs to the precise partitioning and transformation of carbon are still lacking. This paper aims to address the critical problem by providing a comprehensive analysis of carbon metabolism mechanisms within traditional iron and steel manufacturing process and rigorously evaluating the accessibility and implications of existing and emerging reduction strategies.

## 2. Materials and Methods

### 2.1. System Description of Steel Manufacturing

**Figure 1:**
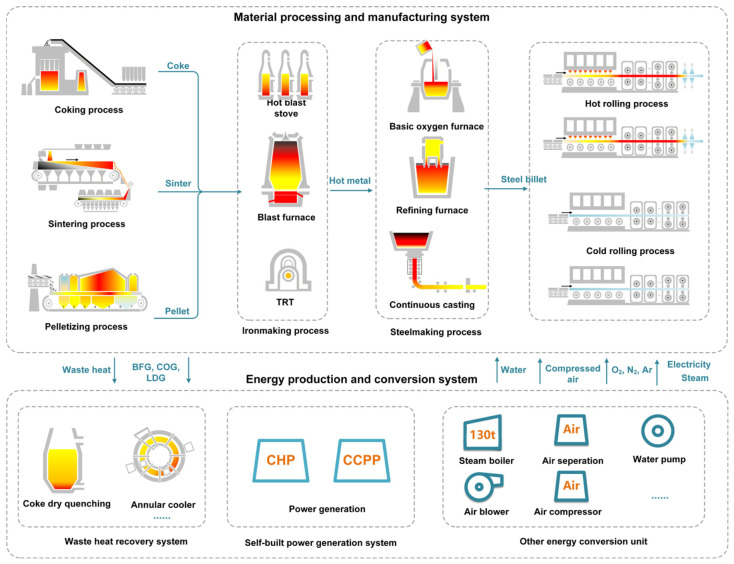
The production system of the BF-BOF manufacturing process.

Figure 1 depicts the system components for an integrated steel production process. The processes and energy production facilities were aggregated nodes rather than individual equipment units, as detailed in Table 1. Each node represented a mass and energy balance boundary within which carbon-containing material inputs, carbon-containing outputs, and indirect energy consumption were quantified. The upstream material processing and manufacturing subsystem comprised coal thermal pyrolysis (coking process) and ore processing (sintering and pelletizing). The ironmaking process, including hot blast stoves, BF, and top gas recovery, converted iron ore, coal, coke, and auxiliary flux into hot metal (HM), by-product gas, and slag. The primary steelmaking subsystem encompassed the BOF, secondary refining furnaces and continuous casting machine, which is responsible for transforming HM into refined molten steel with targeted chemical composition and producing steel slab, while removing impurities such as carbon, sulfur, and phosphorus. The subsequent rolling lines (including hot rolling and cold rolling) further process through thermomechanical deformation and microstructure evolution, producing final steel products such as steel billets and coils. The auxiliary energy production and conversion subsystems mainly include waste heat recovery units, combined heat and power unit (CHP), combined cycle power plant (CCPP), steam boilers, air separation units, compressed air systems, air blowers, and water pumps. The energy subsystem was explicitly incorporated because energy interactions between material manufacturing and energy production can significantly influence the overall emission accounting of iron and steel plant. The production and transportation of upstream materials, including coal, fluxes, and other purchased raw materials, were not included in the system boundary. However, the carbon contained in these materials was included once the materials entered the boundary. The purchased electricity was included through the regional grid emission factor. In addition, emissions of downstream product use, recycling, and disposal were not included in the defined boundary.

### 2.2. Carbon Metabolism Mechanisms in Iron and Steel Production

The core mechanism of the coking process is high-temperature dry distillation of coal in an inert atmosphere under intense heating, which transforms raw coal into distinct carbon-containing products through thermally driven molecular transformations. The main carbon reactions in the coking process are shown in Table A1. The input carbon derived from coking coal enters the oven chamber predominantly in the form of complex macromolecules. Weak chemical bonds in the coking coal break first, releasing primary volatile components such as light aliphatic hydrocarbons. Subsequently, the remaining aromatic structures undergo progressive polycondensation and elimination of hydrogen, oxygen, and nitrogen, forming larger polycyclic aromatic clusters that gradually solidify into the carbon skeleton of coke. The volatile components are gradually released and undergo secondary pyrolysis and reforming in the gas phase, generating crude benzene, hydrogen, methane, carbon monoxide, and other key components of coke oven gas. A small fraction of carbon condenses into high-molecular aromatic compounds, which constitute coal tar. During the coal pyrolysis process, no oxidation or reduction of carbon occurs, and the carbon only undergoes molecular rearrangement and partitioning between solid, liquid, and gaseous phases. Thus, from a decarbonization perspective, the coking process is less about carbon conversion and more about carbon redistribution. In addition, carbon in the combustion chamber is primarily converted through the oxidation of gaseous carbon-containing substances in the fuel gas rather than through the direct participation of solid carbon. The main carbon transformation process involves the combustion of components such as CO, CH_4_, and light hydrocarbons present in blast furnace gas (BFG) and coke oven gas (COG).

Carbon conversion in the sintering and pelletizing process is accompanied by two distinct carbon release pathways, including carbon oxidation, exothermic combustion for thermal energy supply, and thermal decomposition of carbonates in fluxes. The main carbon reactions in the sintering process are shown in Table A2. The input carbon from coal and coke powder is initially present as elemental carbon and undergoes oxidation to CO and CO_2_ via reaction with oxygen in the auxiliary combustion air, releasing the thermal energy required for iron ore agglomeration. Some carbon is chemically bound in carbonate structures in limestone, dolomite, and other fluxes, which is released exclusively through thermal decomposition reactions at high temperatures and causes process emission. This source of carbon is particularly challenging for decarbonization because it is process-inherent and cannot be avoided by substituting fuels. Moreover, the decomposition reactions are strongly endothermic, consuming thermal energy from fuel combustion.

The ironmaking process involves a complex carbon metabolic process, characterized by the circulating oxidation and reduction of carbon within the BF. The main carbon reactions in the ironmaking process are shown in Table A3. The input carbon from coke and injected coal first enters the tuyere combustion zone, reacting with oxygen in hot blast to form CO_2_. Subsequently, the CO_2_ immediately reacts with excess carbon to produce CO, completing the primary carbon transformation. The formed CO rises through the furnace and sequentially reduces iron oxides, including Fe_2_O_3_, Fe_3_O_4_, and FeO, being oxidized to CO_2_ in this process. The CO_2_ encounters descending coke and is converted to CO again, forming a continuous internal carbon oxidation and reduction cycle. Part of the input carbon dissolves directly into HM, and the remaining carbon is converted to CO and CO_2_ as the main component in BFG.

The main function of steelmaking process is the precise control of dissolved carbon in HM. The main carbon reactions in the steelmaking process are shown in Table A4. High-velocity oxygen jets impinge on the surface of molten steel in BOF, forming localized high-temperature reaction zones, where dissolved carbon reacts directly with oxygen to form CO and CO_2_. The majority of carbon is oxidized to CO, which evolves from molten steel as gaseous byproduct. A portion of the evolved CO undergoes secondary combustion to CO_2_ in the furnace headspace, while the remaining CO is recovered by the gas collection system. A small residual amount of carbon remains dissolved in the final molten steel, with its concentration tailored to meet the specifications of different steel grades in secondary refining process.

Carbon pricing policies and CO_2_ emission fees are increasingly transforming carbon emissions from an environmental property into explicit production cost. Climate policy tools such as the EU Carbon Border Adjustment Mechanism and other emerging national carbon markets require steel producers to quantify emissions more accurately and to identify cost-effective mitigation priorities. As the largest steel producer, China has included the iron and steel sector in China Carbon Emissions Trading Exchange (CCETE). For iron and steel enterprises, these policies directly influence strategic decisions on low-carbon transition. The carbon pricing signals fundamentally alter the economic calculation of process carbon management, making the mechanistic understanding of carbon flow patterns directly relevant to operational and investment decisions in steel enterprises.

### 2.3. Carbon Flow and Emission Quantification Method

Carbon exists in various forms within the iron and steel manufacturing process: (a) carbon sources from carbon-containing energy resources, such as coke and injected coal; (b) carbon fixed in products and by-products, including coke, HM, CS, and gases; (c) carbon emitted as flue gas; and (d) indirect released carbon associated with energy consumption, such as electricity and steam. The movement of carbon in a closed loop between production processes is essentially a process of internal resource utilization and transfer. Defining the mechanisms of carbon transfer and absorption within the iron and steel production process allows for the quantification of carbon flow patterns between process nodes, construction of emission attribution, and reallocation of emission responsibilities within the coupled system.

To prevent double counting of carbon, the following rules were applied. (a) When a process supplied carbon-containing by-product gas to other processes or energy facilities for combustion, the carbon from gases was treated as transferred carbon rather than dissipated carbon. The actual emissions were attributed exclusively to the process in which the gas was ultimately consumed, thereby ensuring that the same carbon was counted as dissipated only once at the point of final oxidation. (2) Carbon emissions associated with electricity and steam production were treated as direct emissions at the energy production node. At the consumption nodes, the embodied carbon from energy use was accounted as an indirect contribution rather than being recounted as direct process emission. At the system level, direct and indirect emissions were reported separately to prevent any confusion. (3) Carbon contained in intermediate products was regarded as a carbon input to the downstream process. The resulting CO_2_ emissions were assigned to the downstream process, ensuring that no carbon was counted twice at the upstream process. The internal transferred carbon was counted at the node level to characterize carbon migration, but it was excluded from total external carbon input at the system level.

Within the defined physical boundaries, a material and energy flow network *G* = (*N*, *E*) for the iron and steel production system was constructed. $N\;=\;\left\{n_{i},\;i\;=\;1,\;2,\;\ldots ,\;m\right\}$ represents the physical nodes and energy generation units. $E\;=\;\left\{e_{j},\;j\;=\;1,2,\ldots ,n\right\}$ represents the directed flows between nodes, indicating the paths of material and energy flow. If a production unit consumed carbon-containing substances from an upstream node, it only inherited the corresponding emission attribution for the portion of carbon that was oxidized to greenhouse gases within this process. Conversely, when a process generates carbon-containing products supplied to a downstream node, the emission responsibility for the carbon remaining in the products was decoupled from the source node and transferred to the downstream process where the carbon was ultimately oxidized. For carbon-containing byproducts utilized as energy sources in other processes, emission responsibility was assigned to the process in which these byproducts were oxidized.

Each node can be regarded as a hub for carbon metabolism. A carbon flow matrix $F\;={\;[f_{i,j}]}_{n\times n}$ is defined, where $f_{i,j}\;$ refers to the carbon flow from node *i* to node *j*, and $f_{i,j}=\;0$ indicates no carbon flow between nodes. The carbon input at node $n_{i}$ includes the actual carbon input $C_{\mathrm{INPUT},i}^{\mathrm{ACT}}$ introduced by carbon-containing materials and the implicit carbon flow $C_{\mathrm{INPUT},i}^{\mathrm{VIR}}$ caused by indirect energy consumption. Carbon flows throughout the iron and steel manufacturing system across different constituent nodes strictly adhere to mass conservation principles.

(1) $$\begin{array}{c} C_{\mathrm{INPUT},i}^{\mathrm{ACT}}=C_{\mathrm{OUTPUT},i}^{\mathrm{ACT}} \end{array}$$

The carbon input of node $n_{i}$ can be described as follows:

(2) $$\begin{array}{c} C_{\mathrm{INPUT},i}^{\mathrm{ACT}}=\sum_{n}M_{n,i}^{C}\cdot \omega_{C,n} \end{array}$$

where $M_{n,i}^{C}$ represents the mass of carbon-containing substance *n*, kg; $\omega_{C,n}$ represents the carbon content in substance n, kg/kg.

For a specific flow $e_{j}$, the conversion destination of the attached carbon is often determined by the flow’s functionality, including reduction, heat source, and carrier functions. Accordingly, the actual carbon output can be allocated to distinct destinations following different metabolic pathways.

(3) $$\begin{array}{c} C_{\mathrm{OUTPUT},i}^{\mathrm{ACT}}={\;C}_{\mathrm{FIX},i}+{\;C}_{\mathrm{RT},i}+C_{\mathrm{DIS},i}=\sum_{m}M_{m,i}^{\mathrm{CP}}\cdot \omega_{C,m}+\sum_{n}M_{n,i}^{\mathrm{CT}}\cdot \rho_{C,n}+\sum_{q}M_{q,i}^{\mathrm{DIS}}\cdot \varphi_{C,q} \end{array}$$

where $C_{\mathrm{FIX},i}$ denotes fixed carbon, which represents carbon fixed in products, retained within the iron and steel production system and does not dissipate into the environment, kg; $C_{\mathrm{RT},i}$ refers to transferred carbon, meaning carbon carried as a form of secondary energy and reallocated to downstream nodes, kg; $C_{\mathrm{DIS},i}$ represents dissipative carbon, referring to carbon that is oxidized and ultimately emitted into the atmospheric environment; $M_{m,i}^{\mathrm{CP}}$ represents the mass of product *m* output from node *n_i_*, kg; $M_{n,i}^{\mathrm{CT}}$ represents the secondary energy *n* output from node *n_i_* and transferred to other nodes, kg; $M_{q,i}^{\mathrm{DIS}}$ represents the wastes *q* at node *n_i_*, kg; and $\omega_{C,m}$, $\rho_{C,n}$, and $\varphi_{C,q}$ represent the carbon content in substance *m*, *n*, and *q*, respectively.

Unlike the carbon flow behavior in material flows that strictly follows conservation laws, energy-flow operation does not involve the physical transfer of carbon elements but carries embodied CEs, calculated by consumption and emission factor of energy production. The implicit carbon flow $C_{\mathrm{INPUT},i}^{\mathrm{VIR}}$ of energy system is neither retainable nor transferable and is considered to be instantly and completely dissipated upon entry into the system. The implicit carbon in node *n_i_* is mainly originated from the consumption of key energy carriers, which are mainly converted from electricity and steam.

(4) $$\begin{array}{c} C_{\mathrm{INPUT},i}^{\mathrm{VIR}}=({\mathrm{PC}}_{i}\cdot {\mathrm{CEF}}_{P}+{\;\mathrm{SC}}_{i}\cdot {\mathrm{CEF}}_{S}) \end{array}$$

where ${\mathrm{PC}}_{i}$ and ${\mathrm{SC}}_{i}$ represent the electricity and steam consumption in node *n_i_*, respectively; ${\mathrm{CEF}}_{P}$ and ${\mathrm{CEF}}_{S}$ represent the carbon emission factor of electricity and steam, respectively, kg-C/kW·h or kg-C/kg.

The electricity and steam consumption at node *n_i_* can be expressed as follows:

(5) $$\begin{array}{c} {\mathrm{PC}}_{i}\;=\;{\mathrm{PC}}_{\mathrm{DIR},i}\;+\sum_{m\;=\;1}{\mathrm{EM}}_{m,i}\cdot {\mathrm{EC}}_{m} \end{array}$$

(6) $$\begin{array}{c} {\mathrm{SC}}_{i}={\;\mathrm{SC}}_{\mathrm{DIR},i}+\sum_{m=1}{\mathrm{EM}}_{m,i}\cdot {\mathrm{SC}}_{m} \end{array}$$

where ${\mathrm{PC}}_{\mathrm{DIR},i}$ and ${\mathrm{SC}}_{\mathrm{DIR},i}$ is the direct power and steam consumption in node *n_i_*, respectively, kW·h or kg; ${\mathrm{EM}}_{m,i}$ represents the consumption of energy medium *m* in node *n_i_*, kg or m^3^; and ${\mathrm{EC}}_{m}$ and ${\mathrm{SC}}_{m}$ represents the electricity or steam consumption to produce energy medium *m*.

The comprehensive emission factor of electricity consumed can be described as follows:

(7) $$\begin{array}{c} {\mathrm{CEF}}_{P}\;=\;\frac{12}{44}\sum_{i\;=\;1}^{n}\left(\mu_{i,P}\cdot {\mathrm{CEF}}_{i,P}\right) \end{array}$$

where $\mu_{i,P}$ represents the share of electricity consumption from the power source *i*; ${\mathrm{CEF}}_{i,P}$ is the carbon emission factor of power source *i*, kg-CO_2_/kW·h. Carbon emission factor for power generated by waste heat or energy recovery is set to zero, and the carbon emission factor for purchased electricity is based on the regional grid emission factor. For power plants in steel enterprises, the carbon emission factor of electricity is calculated based on the fuel composition.

The comprehensive emission factor of steam can be described as follows:

(8) $$\begin{array}{c} {\mathrm{CEF}}_{S}\;=\;\frac{12}{44}\sum_{i\;=\;1}^{n}\left(\mu_{i,S}\cdot {\mathrm{CEF}}_{i,S}\right) \end{array}$$

where $\mu_{i,S}$ represents the share of steam consumption from steam source *i*; ${\mathrm{CEF}}_{i,S}$ is the carbon emission factor of power source *i*, kg-CO_2_/kg. Carbon emission factor for steam generated by waste heat recovery is 0 kg-CO_2_/kg. For power plants and steam boilers in steel enterprises, the emission factor of steam can be calculated by fuel combustion. When an energy-supply node$\;n_{i}$ generates multiple energy products (such the CHP and air separation unit), an allocation rule must be introduced. For example, the emission factor for the combined heat and power captive power plant can be calculated based on heat allocation.

(9) $$\begin{array}{c} {\mathrm{CEF}}_{\mathrm{CHP},P}=\left(1\;-\;\frac{H_{\mathrm{con}}^{\mathrm{STEAM}}}{H_{\mathrm{con}}^{\mathrm{TOTAL}}}\right)\sum_{i}\left({\mathrm{FC}}_{i,\mathrm{CHP}}\cdot {\mathrm{CEF}}_{i}^{\mathrm{FUEL}}\right) \end{array}$$

(10) $$\begin{array}{c} {\mathrm{CEF}}_{\mathrm{CHP},S}=\frac{H_{\mathrm{con}}^{\mathrm{STEAM}}}{H_{\mathrm{con}}^{\mathrm{TOTAL}}}\sum_{i}\left({\mathrm{FC}}_{i,\mathrm{CHP}}\cdot {\mathrm{CEF}}_{i}^{\mathrm{FUEL}}\right) \end{array}$$

where ${\mathrm{CEF}}_{\mathrm{CHP},P}$ and ${\mathrm{CEF}}_{\mathrm{CHP},S}$ represents the emission factor of power and steam generated in CHP power plant, kg-CO_2_/kW·h and kg-CO_2_/kg; $H_{\mathrm{con}}^{\mathrm{STEAM}}$ is the heat consumption associated with externally supplied steam, kJ; $Q_{\mathrm{con}}^{\mathrm{TOTAL}}$ is the total heat consumption of CHP power generation unit, kJ; ${\mathrm{FC}}_{i,\mathrm{CHP}}$ denotes the consumption of fuel *m*, kg or m^3^/kW·h; and ${\mathrm{CEF}}_{i}^{\mathrm{FUEL}}$ is the carbon emission factor for fuel m, kg-CO_2_/kWh.

The assignment of zero-emission factors for electricity and steam generated from waste heat recovery are consistent with treatment of by-product energy in existing framework, which can be attributable to plant-level boundary assumption. Waste heat recovery involves energy cascading within the system boundary rather than external energy input. This means that no additional fuel combustion is required to generate the energy carriers within the defined boundary. Waste heat is a by-product energy whose emissions have already been accounted in the upstream industrial processes. Accordingly, attributing additional emissions to the recovery would induce repeated counting. Nonetheless, it should be acknowledged that waste heat recovery can be treated as emission reduction credits under certain carbon credit mechanisms. The effective emission reduction is equal to the marginal emission of the displaced energy.

For CHP units that produce electricity and steam simultaneously, fuel-related emissions are allocated according to the universal allocation method including power bonus method, power loss method, exergy method, alternative generation method, and exergy method. The analysis for different allocation method shows that exergy and power bonus method generally tend to shift allocation factor to electricity production, whereas other are generally more moderate such as energy method [42].

In addition, average and marginal emission factors fundamentally affect the assessment of indirect carbon emissions. The average electricity emission factor reflects the average emission intensity of electricity supplied over a specified period, which is suitable for static carbon accounting and comparison of emissions performance. In contrast, the marginal electricity emission factor represents the change in carbon emissions associated with an incremental change in electricity demand or supply and is thus more appropriate for dynamic assessments of emission impacts resulting from variations in power supply. Therefore, average emission factors are calculated in this study.

The carbon fixation rate (CFR) of process node $n_{i}$ quantifies the capability to retain carbon within material flows without oxidative dissipation. A higher fixation rate indicates a stronger intrinsic capacity of the process node to suppress emissions.

(11) $$\begin{array}{c} {\mathrm{CFR}}_{i}=\frac{C_{\mathrm{FIX},i}}{C_{\mathrm{INPUT},i}^{\mathrm{ACT}}} \end{array}$$

The carbon transfer ratio (CTR) of process node $n_{i}$ characterizes the intensity of carbon conversion from material carriers to secondary-energy carriers as it is transmitted and utilized across processes.

(12) $$\begin{array}{c} {\mathrm{CTR}}_{i}=\frac{C_{\mathrm{RT},i}}{C_{\mathrm{INPUT},i}^{\mathrm{ACT}}} \end{array}$$

The carbon dissipation rate (CDR) directly reflects the node’s environmental burden.

(13) $$\begin{array}{c} {\mathrm{CDR}}_{i}=\frac{C_{\mathrm{DIS},i}}{C_{\mathrm{INPUT},i}^{\mathrm{ACT}}} \end{array}$$

To quantify the relative significance of energy mediums in CE reduction, the contribution rate of energy on carbon intensity for node $n_{i}$ is defined as follows, which can quantify the extent of external contributions to the carbon emission of a specific node.

(14) $$\begin{array}{c} {\mathrm{CRECI}}_{i}=\frac{C_{\mathrm{ENERGY},i}}{C_{\mathrm{ENERGY},i}+{\;C}_{\mathrm{DIRECT},i}} \end{array}$$

where $C_{\mathrm{ENERGY},i}$ represents the implicit (embodied) carbon output attributable to energy consumption, kg; $C_{\mathrm{DIRECT},i}$ represents the direct carbon output of node $n_{i}$, kg.

The terminal carbon dissipation of the entire iron and steel production system can be expressed as follows:

(15) $$\begin{array}{c} C_{\mathrm{DIS},\mathrm{ISMP}}\;=\sum_{i\;=\;1}\varphi_{i}\cdot (C_{\mathrm{DIS},i}\;+\;C_{\mathrm{INPUT},i}^{\mathrm{VIR}}) \end{array}$$

where $C_{\mathrm{DIS},\mathrm{ISMP}}$ refers to the mass of carbon of iron and steel production system emitted into the atmosphere, kg/t-steel; $\varphi_{i}$ represents the steel ratio coefficient of process $n_{i}$, kg/kg.

The proposed carbon metabolism framework differs from conventional methods, as shown in Table 2. The analysis in this study is conducted at the process-node level based on actual plant data with explicit carbon partitioning, whereas conventional carbon flow analysis and IPCC methods operate at the facility or sector level. Conventional approaches mainly quantify total emissions under a predefined boundary, whereas the carbon metabolism framework classifies the carbon distribution at each process node into fixation, transfer, and dissipation types. This classification distinguishes carbon retained in products or byproducts, carbon migration through intermediate material carriers, and carbon finally released to the atmosphere. The traditional approaches treat material and energy flows independently, and modeling for complex energy interconnections is relatively simplified. In addition, uniform predetermined emission factors of energy and materials are employed, which fail to adequately account for the operational discrepancy among enterprises, thereby affecting the accurate assessment of their actual emission. This framework couples carbon-containing material flows with indirect embodied carbon associated with energy mediums, which allows for indirect energy-driven emission responsibility to be identified even for processes with limited direct carbon combustion. This study accounted for the coupling of multiple energy carriers and attributed the associated multi-energy carbon emissions to the end-use consumption of electricity and steam, complemented by energy consumption carbon tracing at the process and node levels. The indicators CFR, CTR, CDR, and CRECI were proposed not only for emission quantification but also for identifying process-specific carbon metabolic pattern. Consequently, this framework provided indirect emission attribution at each process node, enabling targeted decarbonization strategies. In summary, material-flow carbon tracing was coupled with energy-related carbon flows in a unified metabolic model for steel manufacturing, resolving the disconnect between precise material and energy emission accounting. Process-level classification and comparison of carbon metabolic patterns were conducted, providing a basis for matching specific decarbonization technologies to individual process.

### 2.4. Data Resources

Data adopted in this study were derived from actual production data as well as relevant national standards, databases, and literature. The core production and operation data were collected from a representative integrated iron and steel enterprise in North China with a production capacity of approximately 9 million tons of CS annually, covering the complete production chain from raw material processing to steel products. This enterprise adopted the conventional BF-BOF production route, with an equipment configuration that was representative of this type of production process. The steel plant was equipped with four coke ovens with an annual capacity of 4.2 million tons, two sintering machines with an annual capacity of 11 million tons, one belt type roasting machine with an annual capacity of 4 million tons, two 5500 m^3^ BFs, and five 300-ton BOFs. The data represented averaged operating levels under stable production conditions, which were collected at monthly intervals. The data from individual device were aggregated into whole-branch plant primarily because the plant-level production dataset was reported by production process rather than by individual equipment. For example, the data of steelmaking plant covered BOF, secondary refining, and continuous casting. Direct carbon transformation was mainly associated with BOF decarburization reactions, whereas secondary refining and continuous casting contribute to emissions primarily through energy consumption. The input–output parameters of main production processes in iron and steel manufacturing system are depicted in Table 3. The input–output data of the auxiliary energy production facilities are shown in Table 4. The main composition data of consumed gas are shown in Table 5.

## 3. Results

### 3.1. Data Validation

To evaluate the credibility of the calculated results, the result validation is firstly conducted against published benchmarks, as shown in Table 6. The carbon emission is compared with published calculation values of emission intensity for BF-BOF steel production. The existing reported results indicate that the emission intensities exceed 1.8 t-CO_2_/t-CS for the conventional BF–BOF route under broader boundary definitions that include upstream raw-material preparation, external transportation, and downstream product-use stages. The plant-level emissions calculated in this study are generally consistent with reported values within an acceptable range, though slightly lower due to differences in system boundary definitions. The process-level emissions, including coking, ironmaking, steelmaking, and hot rolling, are consistent with reported values. The sintering, pelletizing, and cold rolling processes exhibit relatively larger deviations, mainly due to discrepancies among different enterprises in boundary definitions, fuel compositions, and material and product structure. Overall, the proposed method provides relatively reasonable plant-level and process-level emission assessment when accounting for boundary and structure differences.

### 3.2. Carbon Flow Analysis

In addition, the carbon flow maps based on material and energy flows are constructed in this section to comprehensively explore the migration and transformation patterns of carbon (including physical carbon flows embedded in material transfers and indirect implicit carbon flows associated with energy consumption), thereby supporting subsequent CE analysis and the formulation of decarbonization pathways.

**Figure 2:**
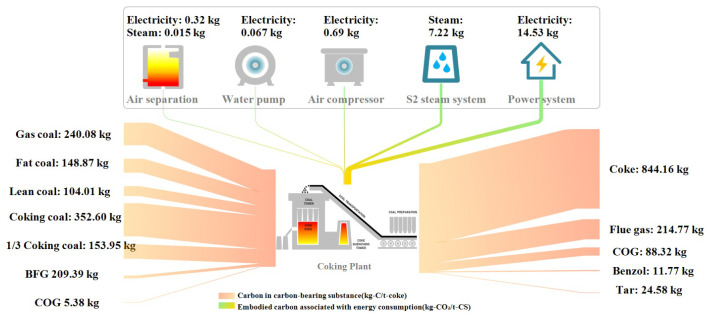
Carbon flow diagram of coking process.

The carbon flow map of the coking process is shown in Figure 2. The total carbon input to the coke oven is 1214.38 kg/t-coke, including 999.61 kg/t-coke from coking coal and 214.77 kg/t-coke from mixed gas, accounting for 82.31% and 17.69% of the total carbon input, respectively. Carbon outputs in coke, flue gas, COG, and other by-products (tar and crude benzene) are 844.16, 214.77, 88.32, and 36.35 kg/t-coke, accounting for 71.32%, 18.15%, 7.46%, and 3.07% of the total carbon output, respectively. The coke oven achieves a CFR, CTR, and CDR of 72.51%, 7.27%, and 17.69%, respectively. Carbon emission attributable to indirect energy consumption is 6.23 kg-C/t-CS (22.84 kg-CO_2_/t-CS), with electricity consumption contributing 68.33% and steam consumption contributing 31.67%, which leads to a CRECI for the coking process of 8.03%.

**Figure 3:**
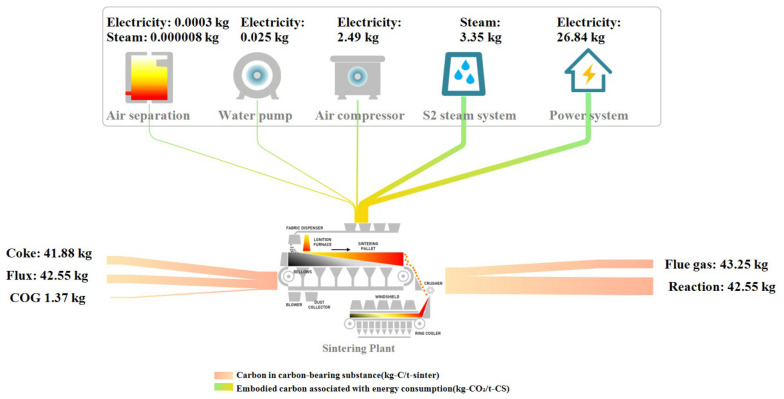
Carbon flow diagram of sintering process.

The carbon flow map of the sintering process is shown in Figure 3. The total carbon input to the sintering machine is 85.80 kg/t-sinter, including 41.88 kg/t-sinter from coke, 1.37 kg/t-sinter from COG, and 42.55 kg/t-sinter from fluxes, accounting for 48.84%, 1.60%, and 49.59% of the total input, respectively. The carbon output includes 43.25 kg/t-sinter from flue gas and 42.55 kg/t-sinter from CaCO_3_ decomposition, accounting for 50.41% and 49.58% of the total carbon output, respectively. The CFR, CTR, and CDR of the sintering process are 0%, 0%, and 100%, respectively. In addition, indirect energy consumption leads to an associated carbon emission of 8.92 kg-C/t-CS (32.71 kg-CO_2_/t-CS), with electricity and steam consumption contributing 89.76% and 10.24%, respectively. The CRECI of the sintering process is 13.07%.

**Figure 4:**
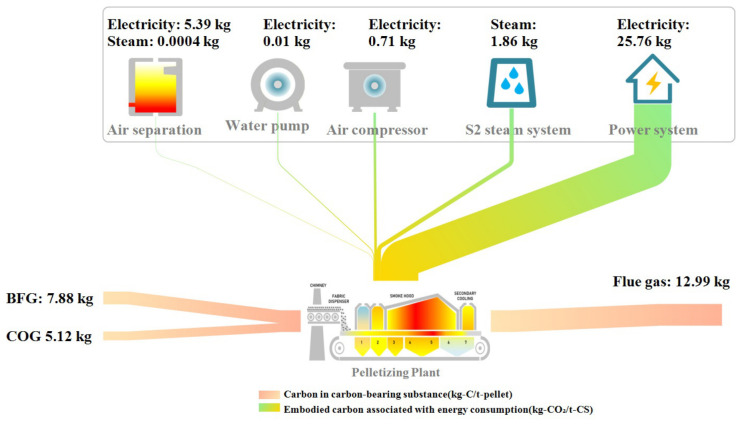
Carbon flow diagram of pelletizing process.

The carbon flow map of the pelletizing process is shown in Figure 4. The pellet production process is characterized by a low carbon intensity. The total carbon input reaches 12.99 kg/t-pellet, including 7.87 kg/t-pellet from BFG and 5.12 kg/t-pellet from COG, accounting for 60.60% and 39.40% of the total carbon input, respectively. The carbon output is 12.99 kg/t-pellet, emitted with flue gas. The pelletizing process has a CFR, CTR, and CDR of 0%, 0%, and 100%, respectively. The indirect energy consumption results in an associated carbon output of 9.20 kg-C/t-CS (33.73 kg-CO_2_/t-CS), with electricity accounting for 94.49%, while steam accounting for only 5.51%. The CRECI of the pelletizing process is 47.58%.

**Figure 5:**
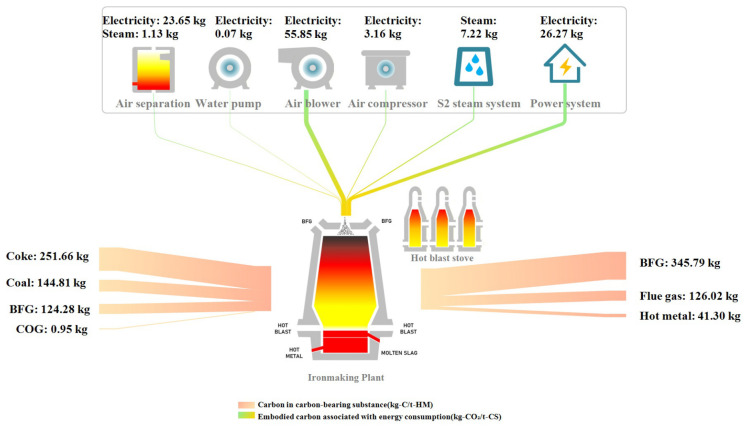
Carbon flow diagram of ironmaking process.

The carbon flow map of the ironmaking process is shown in Figure 5. The total carbon input of the BF is 561.14 kg/t-HM, including 291.45 kg/t-HM from coke, 144.81 kg/t-HM from injected coal, and 125.18 kg/t-HM from gaseous fuels, accounting for 51.91%, 25.79%, and 22.29% of the total input, respectively. The principal carbon outputs include 41.30 kg/t-HM in HM, 345.79 kg/t-HM in BFG, and 126.98 kg/t-HM in flue gas, representing 8.03%, 67.26%, and 24.70% of total carbon output, respectively. The CFR, CTR and CDR of ironmaking process are respectively 7.36%, 61.59%, and 22.62%. The extremely CFR primarily results from the role of carbon as reducing agent and thermal source rather than as a product constituent. Meanwhile, the ironmaking process exhibits a high CTR. To maintain a reducing atmosphere in the BF, excess input carbon is converted to CO and exits in BFG, which is transferred to downstream processes as gas fuel rather than being directly dissipated. Indirect energy utilization results in an associated carbon output of 32.00 kg-C/t-CS (117.35 kg-CO_2_/t-CS), with electricity and steam consumption contributing 92.88% and 7.12%, respectively. The CRECI of the ironmaking process is 20.43%.

**Figure 6:**
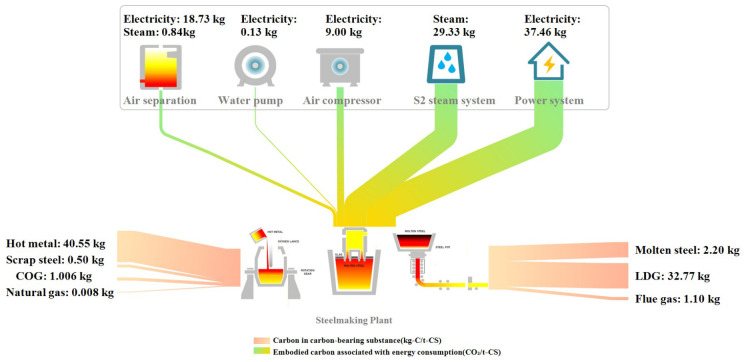
Carbon flow diagram of steelmaking process.

The carbon flow map of the steelmaking process is shown in Figure 6. The total carbon input in BOF is 42.06 kg/t-CS, comprising 40.55 kg/t-CS from HM, 0.50 kg/t-CS from scrap steel, and 1.01 kg/t-CS from gaseous fuels, accounting for 96.41%, 1.19%, and 2.40%, respectively. Major carbon outputs include 2.20 kg/t-CS contained in CS, 32.77 kg/t-CS contained in Linz–Donawitz gas (LDG), and 1.10 kg/t-CS released via flue gas, accounting for 6.10%, 90.85%, and 3.05% of the total carbon output, respectively. The CFR, CTR, and CDR for the steelmaking process are 5.23%, 77.93%, and 2.62%, respectively. Carbon output associated with indirect energy consumption is 26.04 kg-C/t-CS (95.49 kg-CO_2_/t-CS), with electricity and steam consumption contributing 68.41% and 31.59%, respectively. CRECI for the steelmaking process reaches 95.94%.

**Figure 7:**
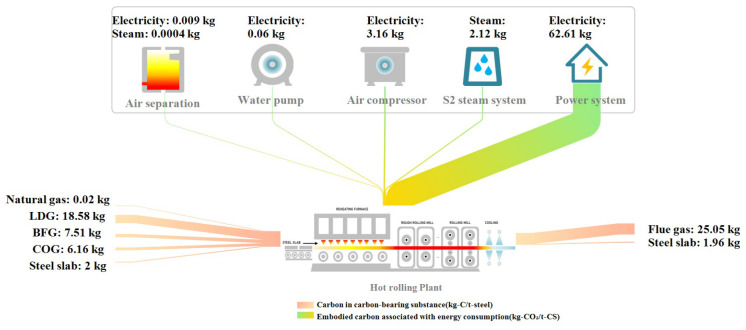
Carbon flow diagram of hot rolling process.

The carbon flow map of the hot rolling process is shown in Figure 7. The total carbon input of the hot rolling process is 34.27 kg/t-SB, including 32.27 kg/t-SB from gas fuel and 2.00 kg/t-SB from SB, accounting for 94.16% and 5.84% of the total input, respectively. The carbon outputs include 1.96 kg/t-SB contained in the SB and 25.05 kg/t-SB carried by flue gas, representing 7.26% and 92.74% of the total output, respectively. The CFR, CTR, and CDR of hot rolling process are 5.72%, 0%, and 73.09%, respectively. Additionally, indirect energy consumption leads to a carbon output of 18.53 kg-C/t-CS (67.96 kg-CO_2_/t-CS), with electricity and steam consumption contributing 96.88% and 3.12%, respectively. The CRECI of the hot rolling process is 42.52%.

**Figure 8:**
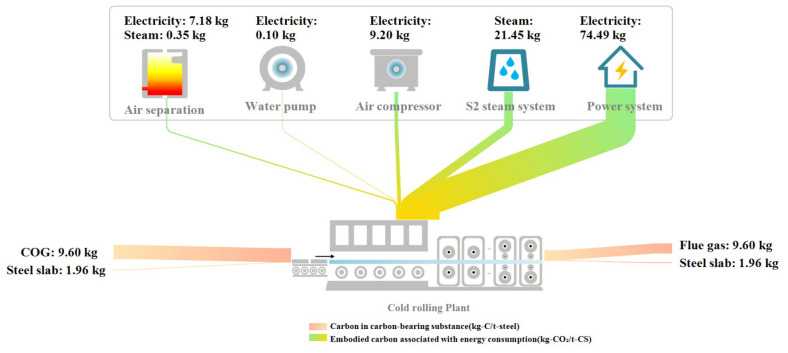
Carbon flow diagram of cold rolling process.

The carbon flow map of the cold rolling process is shown in Figure 8. The total carbon input is 11.56 kg/t-SB, with gas fuel (9.60 kg/t-SB) and SB (1.96 kg/t-SB) accounting for 83.04% and 16.96% of the total, respectively. The CFR, CTR, and CDR of cold rolling process are 16.96%, 0%, and 87.63%, respectively. Unlike processes with high fuel consumption, the cold rolling process exhibits low carbon intensity, with 30.76 kg-C/t-CS carbon output primarily driven by indirect energy consumption (112.77 kg-CO_2_/t-CS). Electricity and steam account for 80.67% and 19.33% of these emissions, respectively. The CRECI of cold rolling process is 76.21%.

**Figure 9:**
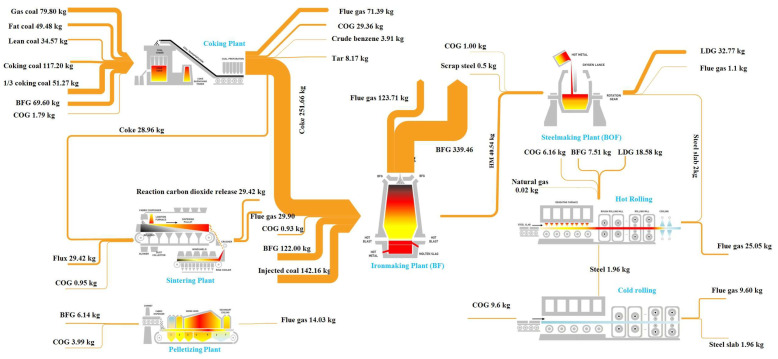
System-level carbon flow diagram of iron and steel production process.

**Figure 10:**
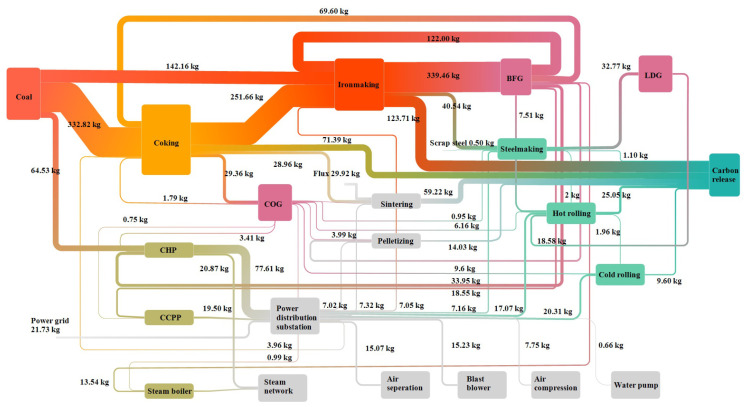
System-level carbon distribution diagram of iron and steel production process.

In addition, the system-level carbon flow distribution diagram is established, as shown in Figure 9 and Figure 10. At the system level, external carbon inputs entering the boundary mainly include coal, fluxes, scrap steel, and other carbon-containing materials. Internal carbon flows, such as coke, HM, CS, COG, BFG, and LDG are treated as carbon transfers between process nodes.

**Figure 11:**
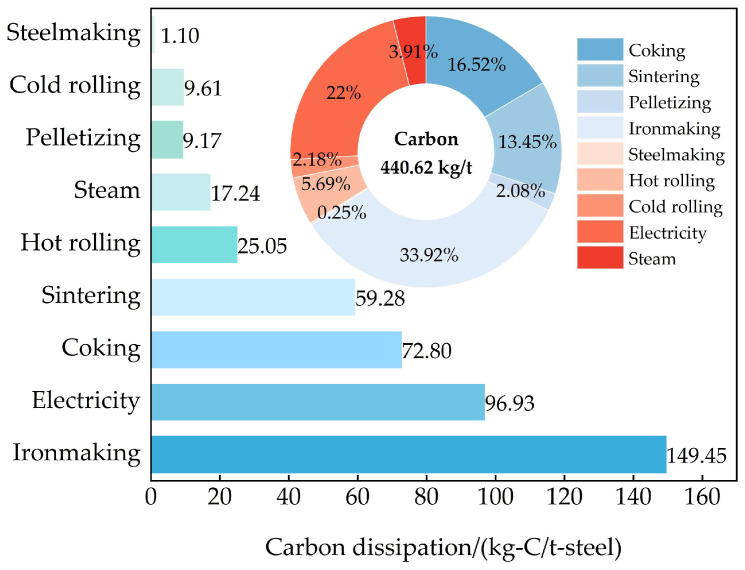
Carbon release intensities of different production units.

The carbon actually released into the atmosphere is compared and analyzed as shown in Figure 11. For the entire steel production system, total carbon dissipation is 440.62 kg-C/t-CS. The ironmaking process shows the highest emissions of 149.45 kg-C/t-CS, accounting for 33.92% of the total and making it the primary target for emission control. Electricity production and consumption contribute 96.93 kg-C/t-CS of carbon release, accounting for 22.00% of the total, which mainly stems from gas and coal combustion and reflects the indirect carbon load of auxiliary equipment. The detailed emission intensity of power and steam consumption of different production processes are shown in Table 7. The sintering process also gives a non-negligible carbon dissipation of 59.28 kg-C/t-CS, arising from solid fuel combustion and carbonate decomposition during the production process. Direct carbon dissipation from the steelmaking and cold rolling process is relatively low, reaching 1.10 and 9.61 kg-C/t-CS, respectively. The low carbon release in cold rolling is mainly attributed to the minimal consumption of gas. For the steelmaking process, although large amounts of CO_2_ are generated, most of the carbon remains in the LDG as a gaseous phase rather than being released directly.

## 4. Discussions

### 4.1. Sensitivity Analysis

**Figure 12:**
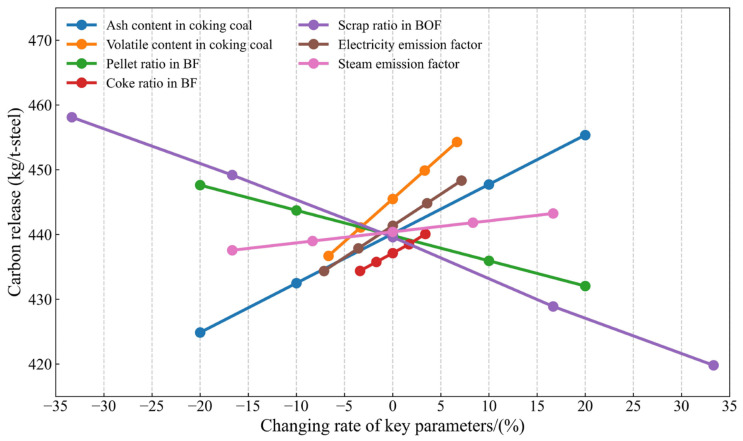
Sensitivity analysis for key influencing factors.

The sensitivity analysis for key influencing factors is conducted to evaluate their influence on total carbon dissipation, as shown in Figure 12. The selected typical parameters include the ash and volatile content in charged coking coal, the pellet ratio, the coke ratio of BF, the scrap ratio in the BOF, and the emission factors for electricity and steam. All the parameters are varied within reasonable intervals above and below their baseline values adopted in this study, derived from actual data uncertainty and literature reports. Various parameters exhibit different fluctuation ranges, depending on the process conditions. When ash content in coking coal, volatile content in coking coal, pellet ratio, coke ratio, scrap ratio, electricity emission factor, and steam emission factor is increased by 1% from its baseline value, the total carbon release increase by 1.12, 1.32, −0.39, 0.84, −0.57, 0.98, and 0.17 t-CO_2_/t-CS, respectively. The results show that total carbon release is most sensitive to the scrap steel ratio and coking coal content. In contrast, variations in steam emission factor have relatively smaller effects on total system-level carbon dissipation. The sensitivity analysis indicates that emission factors, fuel, and material composition are key sources of uncertainty, leading to modest fluctuations in the accounting results.

### 4.2. Carbon Metabolism Mechanisms Analysis

China, India, Japan, the United States, Russia, and Korea are the top six steel manufacturing countries, collectively accounting for over 70% of global CS production [45]. China, Japan, Korea, and Russia exhibit a high self-sufficiency rate of steel product, while North America and many developing economies in Africa and Asia rely significantly on steel imports. Based on a comparison of steel production and imports, the United States, Italy, Mexico, and Turkey show a relatively high degree of reliance on imported steel. This supply–demand geography has important implications for carbon leakage risks under CBAM and similar carbon border mechanisms. Domestic producers in self-sufficient countries may gain competitive advantages in carbon-constrained international markets, whereas the countries depending on imports may face increased steel costs unless their supplying enterprises implement rigorous emission reduction measures. Therefore, accurate carbon accounting and mechanism analysis is not only important for carbon mitigation measures formation but also for low-carbon steel certification, supply-chain carbon management, and carbon cost allocation. The analyses of the carbon metabolism mechanisms and associated mitigation measures for iron and steel production are as follows.

The coking process is dominated by carbon sequestration, with carbon in the charged coal preferentially enriched and immobilized in coke. A relatively small fraction of carbon migrates into COG to supply downstream combustion processes and is directly dissipated to the atmosphere. In summary, the core mechanism of coking is to transfer and fix carbon from raw coal into coke, driven by heat released from combustion of carbon-containing gaseous fuel. As long as high-temperature reactions are maintained, carbon dissipation cannot be avoided. Therefore, the potential for emission reduction mainly stems from source reduction in the coking process, including energy efficiency improvement and energy substitution. The direct carbon dissipation of coke oven (214.77 kg/t-coke) is entirely attributed to the combustion of mixed gas (209.39 kg/t-coke from BFG and 5.38 kg/t-coke from COG) in the heating chamber. One of the most effective carbon reduction measures focuses on reducing fuel consumption, such as high efficiency combustion technology, intelligent combustion optimization and temperature control. In addition, carbon transferred into COG (88.32 kg/t-coke) is predominantly used as fuel for downstream processes, resulting in the shifting of emission responsibility rather than the complete elimination of emissions. Redirecting the utilization of COG from combustion to co-production can fundamentally prevent direct emissions. For example, by reforming COG to produce hydrogen or other products, along with carbon capture and the conversion of by-product CO_2_ for chemical synthesis, significant emission reductions can be achieved. Integrating COG with chemical industries for the production of methanol or ethylene glycol can result in a higher CFR of the coking process.

The sintering process is characterized by an extreme carbon flow pattern dominated by carbon dissipation. Carbon input into the sintering machine (41.88 kg in coke and 1.37 kg in COG) is irreversibly oxidized and released as CO_2_ beyond the sinter machine boundary through flue gas, with no potential for carbon fixation or transfer between processes. Fuel consumption in the sintering machine can be reduced through intelligent batching technology or by enhancing combustion efficiency. Low-carbon flux substitution can eliminate carbonate decomposition emissions, contributing to direct emission reductions. Increasing the pellet ratio in the BF can systematically reduce sinter production, leading to reduction in emissions from the sintering process. Implementing CO_2_ capture and utilization for sintering flue gas can effectively further mitigate emissions, ideally through gas capture before combustion. In summary, the decarbonization of the sintering process relies not only on reducing consumption of solid fuel and energy but also critically on adjusting the process structure.

The direct carbon dissipation and energy-related carbon emissions play almost equally significant roles in the pelletizing process. Acid pellets do not require limestone addition to adjust basicity, thereby avoiding additional CE associated with limestone decomposition. Therefore, compared with the carbon released from gas combustion, carbon reduction in the pelletizing process will rely more on decarbonizing upstream power supply. Meanwhile, optimizing the fuel combustion is also crucial measures for carbon reduction.

The ironmaking process serves as the core carbon metabolic hub of the BF-BOF production route, characterized by a low CFR, high CTR, and moderate CDR. Accordingly, decarbonization of the ironmaking process must simultaneously address direct carbon dissipation control, high-value utilization of transferred carbon, and reconstruction of carbon metabolic pathways, with full consideration of its systemic impacts across the entire production chain. The hot blast stove system accounts for nearly 100% of the direct carbon dissipation in the ironmaking process. This necessitates precise management of its combustion, which can be achieved through advanced combustion technologies to reduce fuel consumption and through intelligent control strategies to enhance gas combustion efficiency. Furthermore, the current utilization pattern of BFG, predominantly as low-grade combustion fuel, can be transformed into high-value-added manufacturing of chemical products such as methanol and synthetic ammonia. Another critical decarbonization pathway is to weaken the carbon-based metallurgical metabolic regime. The thermodynamic role of carbon as a reductant and heat source in traditional BFs inherently dictates the inevitable generation of substantial emissions. Gradually increasing the hydrogen injection ratio in the BF can partially replace coke and injected coal, thereby reducing carbon emission intensity.

In the steelmaking process, 96.41% of the direct carbon input originates from upstream HM, indicating that the majority of carbon is essentially inherited from the ironmaking process rather than generated internally. The carbon introduced by HM from the upstream ironmaking process is largely oxidized and transferred into LDG for downstream utilization, resulting in a high carbon migration rate. In addition, the steelmaking process possesses a distinct energy-oriented emission structure, and its decarbonization potential lies primarily in low-carbon energy transition. Additionally, reducing HM consumption by increasing the scrap steel ratio in BOF is also a key measure for mitigating emissions.

The billets have a minimal carbon content that is further diminished by surface oxidation; therefore, the heating furnace demonstrates a low CFR. The heating furnace primarily generates heat through the combustion of mixed gases from upstream processes, leading to the generation of flue gas and a higher CDR of hot rolling process. The indirect emission effect of the heating furnace is significant, far exceeding direct dissipation. Deep decarbonization of the heating furnace requires a dual approach involving both fuel optimization and the decarbonization of electricity. The cold rolling process represents an extreme case of an energy-driven emission structure. Therefore, the decarbonization of this process will still depend primarily on the decarbonization of energy supply.

The metabolic characterization of carbon flows allows for a functional classification of the different production processes, each entailing differentiated decarbonization priorities. Four typical pathways are identified: (a) structural adjustment, including fuel substitution (hydrogen injection in BF, etc.) and production structure optimization; (b) metabolic pathway restructuring chemical conversion of gases and CO_2_ capture from gases; (c) energy decarbonization such as the deployment of renewable electricity; and (d) energy efficiency enhancement by combustion intensification, process control, and waste heat recovery. The expected emission reduction effect for each strategy is estimated on the basis of the carbon metabolism model, as shown in Table 8.

## 5. Conclusions

This study investigates the carbon metabolism mechanisms within the iron and steel manufacturing process and identifies strategic pathways toward low-carbon production. By developing and applying a carbon metabolism simulation model that comprehensively integrates both material and energy flows, this study enables the precise quantification of carbon fixation, migration, dissipation, and emission contributions across various stages of steel production. The main findings are as follows.

The carbon distribution within the steel production chain is quantitatively characterized. The total carbon dissipation of the integrated iron and steel production system is 440.62 kg-C/t-CS. The ironmaking process is the largest contributor to direct emissions (149.45 kg-C/t-CS), accounting for 33.92% of the total carbon release. Furthermore, indirect carbon emissions attributable to electricity production and consumption reaches 96.93 kg-C/t-CS, contributing 22.00% of the total system carbon dissipation and underscoring the importance of upstream energy decarbonization. The sintering process also exhibits a non-negligible carbon dissipation of 59.28 kg-C/t-CS, primarily originated from solid fuel combustion and carbonate decomposition.

The analysis of specific processes further illustrates diverse carbon metabolic patterns. The coking process demonstrated a high capacity for carbon fixation, with 72.51% of its carbon input being fixed in coke. In contrast, both the sintering and pelletizing processes are characterized by extreme carbon dissipation characteristics, with 0% CFR, 0% CTR, and 100% CDR, indicating that nearly all the input carbon is released into the atmosphere. The ironmaking process shows a low CFR of 7.36%, a high CTR of 61.59%, and a moderate CDR of 22.62%, highlighting its role as a carbon metabolic hub that transfers a substantial portion of carbon to downstream production units. Direct carbon dissipation of steelmaking and cold rolling is relatively lower, reaching 1.10 and 9.61 kg-C/t-steel, respectively, and exhibiting a profound dependency on indirect emissions. The CRECI of the steelmaking and cold rolling process reaches 95.94% and 76.21%, respectively. It indicates that their carbon emissions are overwhelmingly driven by the carbon intensity of energy supply.

The typical low-carbon technologies should be matched with the identified carbon metabolic patterns. Four typical decarbonization pathways are discussed, including structural adjustment, metabolic pathway restructuring, energy efficiency enhancement, and energy decarbonization. The estimated reduction potentials of various decarbonization strategies span a wide range. Higher scrap ratio, hydrogen-rich blast furnace, carbon capture, and green electricity substitution demonstrate considerable emission reduction effects.

## Figures and Tables

**Table 1:** The composition of subprocesses in iron and steel manufacturing system.

Processes or Energy Facilities	Equipment Configuration
Coking	Coke oven, coke dry quenching, gas recovery
Sintering	Raw materials preparation, ignition furnace, sintering machine, annular cooler
Pelletizing	Raw materials preparation, belt type roasting machine
Ironmaking	Hot blast stove, blast furnace, top gas recovery
Steelmaking	Basic oxygen furnace, secondary refining, continuous casting
Hot rolling	Heating furnace, rolling mills
Cold rolling	Annealing furnace, rolling mills
CHP	Two coal–gas co-combustion combined heat and power units
CCPP	Two combined cycle power plant units
Oxygen production	Several air separation units
Blowing unit	Several blast blowers
Air compression	Several air compressors
Boiler	Two gas-fired steam boilers

**Table 2:** The main differences between various carbon analysis methods.

Methods	Focus	Limitation	Carbon Tracing	Indirect Energy Emission
IPCC carbon accounting	Tier method; largely used for macro-level accounting of total emission but with downward compatibility	Limited applicability of emission factors; oversimplified assumptions	Insufficient description of intermediate conversion processes	Uniform predetermined emission factor of multiple energy carriers
LCA	Life-cycle carbon footprint; quantification of environmental impacts	Data quality constraints; insufficient mechanistic mechanisms	Insufficient description of intermediate conversion processes	Uniform emission factor of multiple energy carriers
Traditional carbon flow analysis	Carbon quantity in material flows	Simplification and obscuration of emission contributions from energy production	Carbon transfer in material flows	Carbon tracking of energy is performed only at the production stage, lacking carbon allocation of the demand side.
Mass balance method	Mass balance method	Based on universal mechanistic principles	Detailed carbon flow	Emissions are quantified only for energy production equipment, with no attribution to the consumption side.
This work	Carbon metabolic pattern and emission attribution	Availability of actual data	Node-level carbon allocation	Energy emission attribution to end-use energy forms based on material-energy flow coupling and multi-energy coupling, with energy consumption emissions tracing down to the process and node levels.

**Table 3:** The input–output data of main processes in iron and steel production, kg/t-steel or m^3^/t-steel.

Substance	Coking	Sintering	Pelletizing	Ironmaking	Steelmaking	Hot Rolling	Cold Rolling
Coal	493.08	0	0	176.71	0	0	0
Coke	−332.40	34.28	0	298.12	0	0	0
Flux	0	48.58	4.73	74.99	33.22	0	0
Ore	0	673.14	683.17	73.49	3.93	0	0
Returned ore	0	216.51	0	0	0	0	0
Sinter	0	−690.92	0	690.92	0	0	0
Pellet	0	0	−705.63	705.63	0	0	0
Lime	0	0	0	0	39.23	0	0
Scrap steel	0	0	0	0	155.45	0	0
BFG	277.94	0	21.77	484.65	0	30.19	0
COG	8.64	4.50	17.11	4.23	4.77	29.55	45.473
LDG	0	0	0	0	0	46.80	0
Crude benzene	−4.20	0	0	0	0	0	0
Tar oil	−11.31	0	0	0	0	0	0
Electricity	26.15	39.80	23.38		55.00	84.35	107.40
Steam	29.76	19.13	1.02	4.28	88.50	5.34	69.73
Water	0.39	0.12	0.04	0.34	0.61	0.31	0.50
Nitrogen	2.04	0.001	0.002	46.35	43.92	0.04	35.44
Compressed air	6.24	12.46	3.63	22.47	65.15	13.70	65.60
Blast	0	0	0	830.45	0	0	0
Hydrogen	0	0	0	0	0	0	2.28
Oxygen	0	0	0	78.55	53.38	0.02	0
BF slag ^(r)^	0	0	0	−250.79	0	0	0
BOF slag ^(r)^	0	0	0	0	−88.54	0	0
Hot metal ^(r)^	0	0	0	−981.74	981.74	0	0
Crude steel	0	0	0	0	−1000	1012.15	1012.15
BFG ^(r)^	0	0	0	−1348.68	0	0	0
COG ^(r)^	−141.85	0	0	0	0	0	0
LDG ^(r)^	0	0	0	0	−119.90	0	0
Steam ^(r)^	41.89	41.46	0	0	93.30	98.40	0
Electricity ^(r)^	44.62	0	0	49.83	0	0	0

The superscript (r) denotes the amount of material or energy recovered in the production process, which is marked as a negative value.

**Table 4:** The input–output data of the auxiliary energy production facilities.

Equipment	Item		Unit	Value
CHP	Input	BFG	m^3^	0.41
		COG	m^3^	0.05
		LDG	m^3^	0
		Coal	kg	0.29
		Electricity	kWh	0.06
	Output	Electricity	kWh	1.00
CCPP	Input	COG	m^3^	0.09
		LDG	m^3^	1.90
		Electricity	kWh	0.02
		Nitrogen	m^3^	0.03
	Output	Electricity	kWh	1.00
Boiler	Input	BFG	m^3^	6.60
		COG	m^3^	830.00
		Electricity	kWh	9.00
		Compressed air	m^3^	0.98
	Output	Steam	t	1.00
O_2_ production	Input	Electricity	kWh	0.78
		Steam	kg	0.09
	Output	Oxygen	m^3^	1.00
Blower	Input	Electricity	kWh	0.09
	Output	Blast	m^3^	1.00
Air compressor	Input	Electricity	kWh	0.14
	Output	Compressed air	m^3^	1.00

**Table 5:** The main component of gases.

Component	CO	CO_2_	CH_4_	H_2_	N_2_	C_2_H_4_	O_2_
BFG	25.40	22.30	0	4.00	49.40	0	0
COG	6.20	2.20	26.00	58.00	4.50	2.50	0.60
LDG	55.00	20.00	0	1.00	23.00	0	0.60

**Table 6:** Validation for direct carbon emission results (kg-CO_2_/t-steel).

Nodes	IPCC [43]	LCIL [43]	Zhang et al. [17]	Zhang et al. [44]	Yuan et al. [8]	This Work	Unit
Coking	-	-	770.32	-	748.71	803.10	kg-CO_2_/t-coke
Sintering	-	153.00	224.82	269.93	258.10	314.38	kg-CO_2_/t-sinter
Pelletizing	-	50.00	77.71	43.21	71.95	43.12	kg-CO_2_/t-pellet
Ironmaking	-	-	-	686.81	500.58	558.19	kg-CO_2_/t-HM
Steelmaking	-	21.00	-	5.11	4.29	4.04	kg-CO_2_/t-CS
Hot rolling	-	-	186.16	64.52	113.84	91.86	kg-CO_2_/t-steel
Cold rolling	-	-	-	11.16	19.31	35.24	kg-CO_2_/t-steel
Electricity	-	-	-	296.14	313.02	355.41	kg-CO_2_/t-steel
Plant	1717	1715	1689.54	1678	1891.92	1615.67	kg-CO_2_/t-steel

**Table 7:** The emission intensity of power and steam consumption in production processes.

Process	Emission of Power	Emission of Steam	Total Energy Emission	Unit
Coking	15.61	7.23	22.84	kg/t coke
Sintering	29.36	3.35	32.71	kg/t-sinter
Pelletizing	31.87	1.86	33.73	kg/t-pellet
Ironmaking	108.99	8.36	117.35	kg/t-HM
Steelmaking	65.32	30.17	95.49	kg/t-CS
Hot rolling	65.84	2.12	67.96	kg/t-SB
Cold rolling	112.77	90.97	21.80	kg/t-SB

**Table 8:** The emission reduction effects for typical decarbonization strategies relative to the current baseline [10,46,47].

Num.	Technology	Key Conditions	Reduction Potential (kg-C/t-steel)
T1	High pellet ratio in BF	The pellet ratio in BF is increased from 40% to 60%	15.57
T2	High scrap ratio in BOF	The scrap ratio in BF is increased from 10% to 20%	38.30
T3	Hydrogen injection in blast furnace	Hydrogen injection can reduce fuel ratio up to 188.60 kg/t-HM; coke replacement ratio is 0.53	30.37
T4	COG injection in blast furnace	COG injection can reduce fuel ratio up to 111.3 kg/t-HM; coke replacement ratio is 0.18	31.92
T5	CCS on oxygen BF (TGR-CCS)	The emission reduction ratio reaches 36%	34.89
T6	Air-blown BF with CCS (BF-CCS)	The emission reduction ratio reaches 17%	16.48
T7	Green electricity substitution	30% green power substitution ratio	29.08
T8	COG to methanol (oxygen-steam reforming method)	The emission reduction ratio reaches 16.7 kg/t-steel	4.57
T9	High temperature–pressure coke quenching	The CO_2_ emission reduction is 35.80 kg/t-coke	3.25
T10	Coke oven carbonization chamber waste gas recovery and automatic pressure adjustment	The CO_2_ emission reduction is 15.40 kg/t-coke	1.40
T11	Thick layer sintering	The CO_2_ emission reduction is 3.90 kg/t-sinter	0.74
T12	Dehumidification technology of blast in BF	The CO_2_ emission reduction is 30.20 kg/t-HM	8.09
T13	Waste heat recovery of slag flushing water	The CO_2_ emission reduction is 28.80 kg/t-HM	7.71
T14	Oxygen-enriched combustion in hot rolling	The emission reduction ratio reaches 15.20%	3.81

## Data Availability

The original contributions presented in this study are included in the article. Further inquiries can be directed to the corresponding author.

## Abbreviations

The following abbreviations are used in this manuscript:

BF	Blast furnace
BOF	Basic oxygen furnace
EAF	Electric arc furnace
CHP	Combined heat and power plant
CCPP	Combined cycle power plant
CFR	Carbon fixation rate
CTR	Carbon transfer ratio
CDR	Carbon dissipation rate
CRECI	Contribution rate of energy on carbon intensity
BFG	Blast furnace gas
COG	Coke oven gas
LDG	Linz–Donawitz gas
HM	Hot metal
CS	Crude steel
SB	Steel billet

## Appendix A

**Table A1:** The main carbon reactions in the coking process.

No.	Reaction Type	Reaction Equation
R_1_	Carbon oxidation reaction	$2\mathrm{CO}+O_{2}=2{\mathrm{CO}}_{2}$
R_2_	Carbon oxidation reaction	${\mathrm{CH}}_{4}+2O_{2}={\mathrm{CO}}_{2}+2H_{2}O$
R_3_	Carbon oxidation reaction	$2C_{2}H_{6}+7O_{2\;}=4{\mathrm{CO}}_{2}+6H_{2}O$
R_4_	Carbon oxidation reaction	$C_{2}H_{4}+3O_{2}=2{\mathrm{CO}}_{2}+2H_{2}O$
R_5_	Thermal pyrolysis reaction	$C_{2}H_{6}=C_{2}H_{4}+H_{2}$
R_6_	Thermal pyrolysis reaction	$C_{2}H_{4}=C+{2H}_{2}$
R_7_	Thermal pyrolysis reaction	${\mathrm{CH}}_{4}=C+2H_{2}$

**Table A2:** The main carbon reactions in the sintering process.

No.	Reaction Type	Reaction Equation
R_1_	Carbon oxidation reaction	$CO+0.5O_{2}={\mathrm{CO}}_{2}$
R_2_	Carbon oxidation reaction	${\mathrm{CH}}_{4}+2O_{2}=CO_{2}+2H_{2}O_{\left(g\right)}$
R_3_	Carbon oxidation reaction	$C+O_{2}=CO_{2}$
R_4_	Carbon oxidation reaction	$2C+O_{2}=2\mathrm{CO}$
R_5_	Redox reaction	$C+CO_{2}=2CO$
R_6_	Carbon oxidation reaction	$C+{\;H}_{2}O_{\left(g\right)}=CO+H_{2}$
R_7_	Carbon oxidation reaction	$3{Fe}_{2}O_{3}+CO=2{Fe}_{3}O_{4}+CO_{2}$
R_8_	Thermal decomposition reaction	$CaCO_{3}=C\mathrm{aO}+CO_{2}$
R_9_	Thermal decomposition reaction	$C\mathrm{aMg}{\left({\mathrm{CO}}_{3}\right)}_{2}=C\mathrm{aO}+M\mathrm{gO}+2CO_{2}$

**Table A3:** The main carbon reactions in the ironmaking process.

No.	Reaction Type	Reaction Equation
R_1_	Carbon oxidation reaction	$Fe_{2}O_{3}+\mathrm{CO}=2Fe_{3}O_{4}+CO_{2}$
R_2_	Carbon oxidation reaction	$Fe_{3}O_{4}+\mathrm{CO}=3\mathrm{FeO}+CO_{2}$
R_3_	Carbon oxidation reaction	$\mathrm{FeO}+\mathrm{CO}=\mathrm{Fe}+CO_{2}$
R_4_	Redox reaction	$2C+O_{2\;}=2\mathrm{CO}$
R_5_	Carbon oxidation reaction	$C+O_{2}=CO_{2}$
R_6_	Redox reaction	$C+CO_{2\;}=2\mathrm{CO}$
R_7_	Carbon oxidation reaction	$C+H_{2}O=\mathrm{CO}+H_{2}$
R_8_	Carbon oxidation reaction	$\mathrm{CO}+{\;H}_{2}O=CO_{2}+H_{2}$
R_9_	Steam methane reforming	$CH_{4}+H_{2}O=\mathrm{CO}+3H_{2}$
R_10_	Carbon oxidation reaction	FeS+CaO+C=CaS+CO+Fe

**Table A4:** The main carbon reactions in the steelmaking process.

No.	Reaction Type	Reaction Equation
R_1_	Redox reaction	$2C+O_{2}=2\mathrm{CO}$
R_2_	Carbon oxidation reaction	$C+O_{2}=CO_{2}$
R_3_	Carbon oxidation reaction	$2\mathrm{CO}+O_{2}=2CO_{2}$
R_4_	Carbon oxidation reaction	C+FeO=Fe+CO
R_5_	Iron oxidation reaction	$2\mathrm{Fe}+O_{2}=2\mathrm{FeO}$
R_6_	Silicon oxidation reaction	$\mathrm{Si}+2\mathrm{FeO}=\mathrm{Si}O_{2}+2\mathrm{Fe}$
R_7_	Silicon oxidation reaction	$\mathrm{Si}+O_{2}=\mathrm{Si}O_{2}$
R_8_	Manganese oxidation reaction	Mn+FeO=MnO+Fe
R_9_	Manganese oxidation reaction	$2\mathrm{Mn}+O_{2}=2\mathrm{MnO}$
R_10_	Phosphorus oxidation reaction	$2P+5\mathrm{FeO}=P_{2}O_{5}+5\mathrm{Fe}$
R_11_	-	$P_{2}O_{5}+4\mathrm{Ca}O=4\mathrm{Ca}O\cdot P_{2}O_{5}$
R_12_	Sulfur oxidation reaction	FeS+CaO=CaS+FeO
